# Influence of Molting and Starvation on Digestive Enzyme Activities and Energy Storage in *Gammarus fossarum*

**DOI:** 10.1371/journal.pone.0096393

**Published:** 2014-04-30

**Authors:** Laetitia Charron, Olivier Geffard, Arnaud Chaumot, Romain Coulaud, Ali Jaffal, Véronique Gaillet, Odile Dedourge-Geffard, Alain Geffard

**Affiliations:** 1 Université Reims Champagne Ardenne, Unité Interactions Animal-Environnement, Reims, France; 2 Institut national de Recherche en sciences technologiques pour l’environnement et l’agriculture, Unité de Recherche Milieux Aquatiques, Ecologie et Pollutions (MAEP), Villeurbanne, France; Uppsala University, Sweden

## Abstract

Among the many biological responses studied in ecotoxicology, energy-based biomarkers such as digestive enzyme activities and energy reserves appear to be useful predictive tools for detecting physiological disturbances in organisms. However, the use of these biological responses as biomarkers could be limited by the effects of confounding factors (biotic and abiotic) and physiological processes, such as the reproductive cycle. Thus, the optimal use of these biomarkers will be facilitated by understanding the effects of these factors on the energy metabolism of the sentinel species being studied. We considered abiotic factors (temperature and conductivity) in a previous study, whereas the present study investigated the effects of gender, the female reproductive stage, and food availability on the digestive enzyme activities and energy storage of *Gammarus fossarum*. The results indicated that, during the female reproductive cycle, the activities of digestive enzymes (amylase, cellulase, and trypsin) decreased significantly, whereas the levels of reserves (proteins, lipids, and sugar) increased until the last premolt stage. Restricted food diets only led to decreased amylase activities in both sexes. Food starvation also induced a decrease in the energy outcomes in females, whereas there were no effects in males. In general, the biochemical (digestive enzyme activities) and physiological (energy reserves) responses were more stable in males than in females. These results support the use of males fed *ad libitum* to limit the effects of confounding factors when using these energy biomarkers in *Gammarus fossarum* during biomonitoring programs.

## Introduction

A challenge in aquatic ecotoxicology is the development of relevant ecological tools for detecting the roles of chemical contaminations in environmental quality degradation. In general, population level biological impacts can be tracked by monitoring many responses at lower levels of biological organization (sub-individual or individual) because some of them are related more or less directly to fitness traits (survival, reproduction, and growth) [1]. Based on this framework, several ecotoxicological studies have investigated biological responses related to energy metabolism. Early disturbances in energy intake can affect growth, maintenance, reproduction, and energy reserves [2]. Thus, variation in energy metabolism appears to be a good predictive tool for detecting physiological disturbances in organisms that are linked to ecosystem quality [3]. For example, the cellular energy allocation approach is ecologically relevant because cellular effects are linked to higher levels of biological organization in crustaceans [4], [5] and mollusks [6]. Similarly, a decrease in the scope for growth appears to be a sensitive indicator of stress in invertebrates [7], [8]. Variations in energy reserves can also be considered biochemical responses to stress [2]. Previous studies of gammarids have shown that significant modulations in the feeding rates and the activities of digestive enzyme are correlated with chemical water quality [9], [10]. However, the same studies highlighted the potentially strong effects of confounding factors and the importance of characterizing such biological responses accurately before using them as biomarkers.

Chemical stress is not the only source of disrupted responses in organisms and many potential variable factors can lead to misinterpretations [11]. Thus, biomarkers can be influenced by different environmental factors. For many years, studies have focused on the effects of environmental factors as external sources of variability in biological responses [9], [12]. Studies of these external sources of variability have considered many abiotic effects, such as temperature [9], [13], [14], salinity [14], pH [15], and conductivity [9], [13]. The effects of external biotic parameters have also been investigated in biotic relationships such as parasitism [16], [17] and symbiosis [18], [19]. In studies of energy acquisition, the amount of food available must be considered as a major source of variability that affects digestive enzyme activities [20] and energy reserves [21], [22]. In addition, it may be necessary to consider intrinsic biotic characteristics that define the organism being studied. In general, biotic parameters such as gender [23], stage of development [24], sexual maturity, and the reproduction cycle [25] must be considered as factors that are likely to affect biomarkers. Thus, the optimal use of biomarkers will be facilitated by understanding the effects of confounding factors on the biochemistry, physiology, and behavior of the sentinel species being studied.

Among the freshwater species that are used intensively in ecotoxicology, *Gammarus fossarum* is a representative test species. In addition to its sensitivity to contaminants [26], this organism is widespread and has a high population density in the West Palaearctic. Moreover, gammarids play major roles in the European freshwater ecosystem. First, they represent a major source of food for macro-invertebrates, fishes, amphibians, and bird species [27]. Second, gammarids have a key role in the food web as common shredders and they are important for leaf litter breakdown processes [28]. Thus, physiological disturbances in *Gammarus* spp. may have effects at higher levels of biological organization [29].

The present study focused on understanding the physiological processes related to energy metabolism during: (i) the reproductive cycle of females organisms and (ii) starvation. Our previous study analyzed the effects of abiotic factors (temperature and conductivity) [13], whereas the present complementary study investigated the effect of different factors (gender, female reproductive cycle, and food availability) on the activities of digestive enzymes and energy storage in *G. fossarum*. These two approaches may help to elucidate the effects on biomarker responses of parameters that are considered to be confounding factors, thereby facilitating their use in biomonitoring studies.

## Materials and Methods

### Ethics Statement

Gammarids were sampled by members of the Institut National de Recherche en Sciences et Technologies pour l’Environnement et l’Agriculture (IRSTEA), which is the French national institute of science and technology for environment and agriculture. All of the necessary authorizations for sampling were obtained for the designated site at La Tour du Pin.

In France, amphipod research does not require permission, *G. fossarum* is not a protected species and its use in scientific research does not require any specific authorization. All efforts were made to minimize suffering during laboratory experiments.

### Collection and Maintenance of Gammarids

Gammarids were collected by kick sampling at La Tour du Pin, upstream of the Bourbre River (France). This station has good water quality according to the data records of the RNB (French Watershed Biomonitoring Network) and a high density of gammarids. Adult *G. fossarum* were collected using a hand-held net and recovered using 2–2.5 mm mesh sieves. Immediately after sampling, the organisms were kept in a plastic bucket and transferred promptly to the laboratory. The organisms were maintained during a 15-day acclimatization period in 30-L tanks with constant aeration at 14±0.5°C using a 10/14 h light/dark photoperiod. The tanks were supplied continuously with drilled groundwater mixed with softwater (obtained by reverse osmosis) to adjust the sampling site conductivity to 600 µS cm^−1^. The organisms were fed *ad libitum* with alder leaves (*Alnus glutinosa*), which had been conditioned for at least 6±1 days in groundwater. Freeze-dried *Tubifex* sp. worms were added as a dietary supplement twice each week.

### Experimental Design

#### Effects of gender and the female reproductive cycle on energy metabolism parameters

*G. fossarum* is an example of a crustacean where the molting and reproductive cycles can occur concurrently in sexually active females. According to a previous study [30], several features characterize the molt and reproductive stages in females. For *G. fossarum,* Geffard et al. [30] described six molt stages, which are determined by the hardening of the cuticle, the retraction of the epidermis from the cuticle, and the secretion of the new cuticle. The six different stages are referred to as postmolt (A and B), intermolt (C1 and C2), and premolt (D1 and D2). Molting stage A is very short and the two postmolt stages (A and B) are grouped into a single AB stage. The reproductive behavior and oocyte maturity change during the molt cycle. The beginning of the molt cycle (stage AB) is characterized by spawning and fertilization. The first vitellogenesis occurs between stages AB and C1, where the apparent mean follicle surface measures 24800–45100 µm^2^ [30]. The second vitellogenesis occurs between stages C2 and D2, where the follicle surface grows significantly to 106000–164000 µm^2^. Between stages C2 and D2, a male will grasp a female and place her in a position of precopulatory amplexus until the time of fertilization [31].

According to Geffard et al. [30], the female reproductive (and molt) stages can be determined by studying the cuticle shape. Thus, the ends of the third and fourth periopod pairs (dactilopodite and protopodite) in each female were cut using Wecker’s scissors. The periopod pieces were then prepared on a microscope slide with a coverslip and their integumental morphogenesis was observed (×200) to discriminate the five molt stages (AB, C1, C2, D1, and D2). For each moulting stages, six individual specimens were kept to measure their energy reserves and six pools of three specimens were used to measure the activities of digestive enzyme. In all cases, embryos were collected manually from the marsupium and eliminated. All of the samples were weighed, frozen in liquid nitrogen, and stored at –80°C until they were analyzed.

In males, reproductive cycle (spermatogenesis) is little known and documented. Spermatogenesis duration is very short, six days after mating, the maximum stock of sperm in the testes is again obtained. Contrary to females, spermatogenesis is not related to moulting cycle, consequently morphological parameters are not available to accurately sample organism at different spermatogenesis stages. Finally, all males organisms sampled were in amplexus, (associated with females), consequently we are sure that they were in similar spermatogenesis stages (mature). As for females, six males were kept to measure their energy reserves and six pools of three males were used to measure the activities of digestive enzyme.

#### Effects of food availability on energy metabolism parameters

After the acclimatization period, 210 females in the same molt stages (AB) and 210 males were selected for the study. Seven males and seven females were placed in each glass beaker (n = 30) with continuously renewed water (the acclimatization conditions are described above). According to previous experiments (Geffard et al., 2010), we applied feed conditions to induce gradually starvation diet of 0% (control) 50% or 75%. In these controlled conditions (n = 10 glass beakers), the organisms were fed *ad libitum* with discs of alder leaves that measured 2 cm in diameter 7 days each week (these conditions are referred to as 7/7). In two other conditions (n = 10 beakers per condition), the organisms were fed by supplying them with discs of alder leaves only 2 days each week (2/7, corresponding to 50% starvation diet) or only 1 day each week (1/7, corresponding to 75% starvation diet). After each feeding period in the 2/7 and 1/7 conditions, the remaining food was removed from the beakers and kept in a cold room for use in the next meal.

Females associated with males were sampled after 11 and 23 days at stages C1 and D1, respectively, in controlled conditions at 14±0.5°C. For each sampling period (11 and 23 days), each level of food tested (7/7, 2/7, and 1/7), and each gender, six individual organisms were kept to test their energy reserves and six pools of three organisms were collected to measure the activities of their digestive enzyme. All of the samples were weighed, frozen in liquid nitrogen, and stored at –80°C until they were analyzed.

During this experiment, survival rates fluctuated between 93 to 89% and were not significantly different between exposure conditions.

#### Measurement of the digestive enzyme activities

Each pool of gammarids from both experiments was homogenized in Tris-HCl buffer (0.01 M, pH 7) for 2 min using a mixer mill (Retsch) at a frequency of 30 Hz. The homogenate was centrifuged at 10,000×*g* for 10 min at 4°C. The supernatant was collected and stored at −80°C until the analysis.

The carbohydrase activities (cellulase and amylase) were measured using the 3,5-dinitrosalicylic reagent method [32], based on a published protocol [33]. The amylase activity was measured after 30 min of incubation at pH 7 and 42°C with a starch substrate (1%). The cellulase activity was measured after 60 min of incubation at pH 5.5 and 45°C with a carboxymethylcellulose substrate (2%). A calibration curve was established using maltose as the reference sugar. According to the protocol described by Garcia-Carreño and Haard [34], the trypsin activity was measured after 10 min of incubation at pH 8 and 45°C with the substrate of N-benzoyl-DL-arginine 4-nitroanilide hydrochloride (3 mM). A calibration curve was established using p-nitroaniline (p-Na). Each enzyme activity was expressed as micrograms of the final product released per minute and per milligram of protein. The total and cytosolic protein contents in the supernatant were determined according to a published method [35] using bovine serum albumin (BSA) as the protein standard.

#### Measurement of energy reserves

To measure the amounts of total lipids, free sugar, and glycogen in individuals, each organism (n = 6 per condition) was placed in a tube containing 800 µL of methanol and three stainless steel balls. The samples were ground by shaking the tube for 2 min at 30 Hz (Mixer Mill MM 400, Retsch, Haan, Germany). Each homogenized sample was divided into two identical volumes (A and B), where A was used to measure the total lipids and B was used to determine the free sugars and glycogen. The energy reserves were measured according to a method adapted from Plaistow et al. [36].

Chloroform (1∶2 v/v) was added to each A homogenate to solubilize the total lipids. All of the samples were vortexed and kept for 20 min at 4°C. After vortexing, 100 µL of the homogenate was transferred to 16×100 mm culture tubes. The tubes were heated to 95°C in a water bath for 5 min, ensuring that the solvent had evaporated completely. Next, 200 µL of sulfuric acid (95%) was added to each tube before replacing them in the water bath for 10 min at 95°C. The tubes were then removed from the hot water, placed in an ice water bath, and 5 mL of vanillin-phosphoric acid reagent was added. The optical density was measured at 525 nm after 25 min. The quantity of lipids in the samples was determined using a calibration curve based on a standard olive oil (Sigma-Aldrich) solution (1 g/L), which was solubilized in chloroform.

All carbohydrates, including glycogen and free sugars, were determined using homogenate B. To separate the carbohydrates, 200 µL of sodium sulfate solution (2%) was added to the homogenate. After 20 min at 4°C, glycogen was adsorbed onto the sodium sulfate precipitate. The samples were centrifuged for 4 min at 2000×*g* and 4°C to remove the glycogen residues. Next, the supernatant (solution 1) and the pellet were analyzed separately. To measure the glycogen content, the pellet was resuspended in 400 µL of distilled water (solution 2) and three stainless steel balls were added, followed by shaking for 1 min at 30 Hz (Mixer Mill MM 400, Retsch, Haan, Germany). After vortexing, 300 µL of the supernatant (solution 1) containing soluble free sugars and 300 µL (solution 2) of the glycogen homogenate were transferred into separate 16×100 mm culture tubes. Five milliliters of anthrone reagent was added to the tubes, which were then placed in a water bath at 95°C for 17 min. The tubes were removed from the water bath and kept in a cold bath for 10 min to stop the reaction. The optical density of each sample was measured at 630 nm to determine the soluble carbohydrate and glycogen levels. The carbohydrate level was determined by reference to a calibration curve prepared using a standard solution of glucose (1 g/L) in distilled water.

The available energy (Ea) was determined based on the total protein, carbohydrate, and lipid contents at each stage. Each type of energy reserve was transformed into its energetic equivalent using the combustion enthalpy (24000 mJ/mg for protein, 17500 mJ/mg for carbohydrates, and 39500 mJ/mg for lipids) [2].

### Statistical Analysis

The statistical procedures were performed using XLSTAT [37]. The normality and homogeneity of the data were tested first using Shapiro-Wilk and Levene tests. These criteria were not satisfied, so nonparametric tests were used to analyze the data. The Kruskal-Wallis test was used to study the effects of the molt stage or gender on the physiological responses. For the starvation study, the diet effect and time exposure were assessed independently using the Mann-Whitney test.

## Results and Discussion

### Effects of Gender and Female Reproductive Cycle on Energetic Metabolism Biomarkers

#### Females

**Figure 1:**
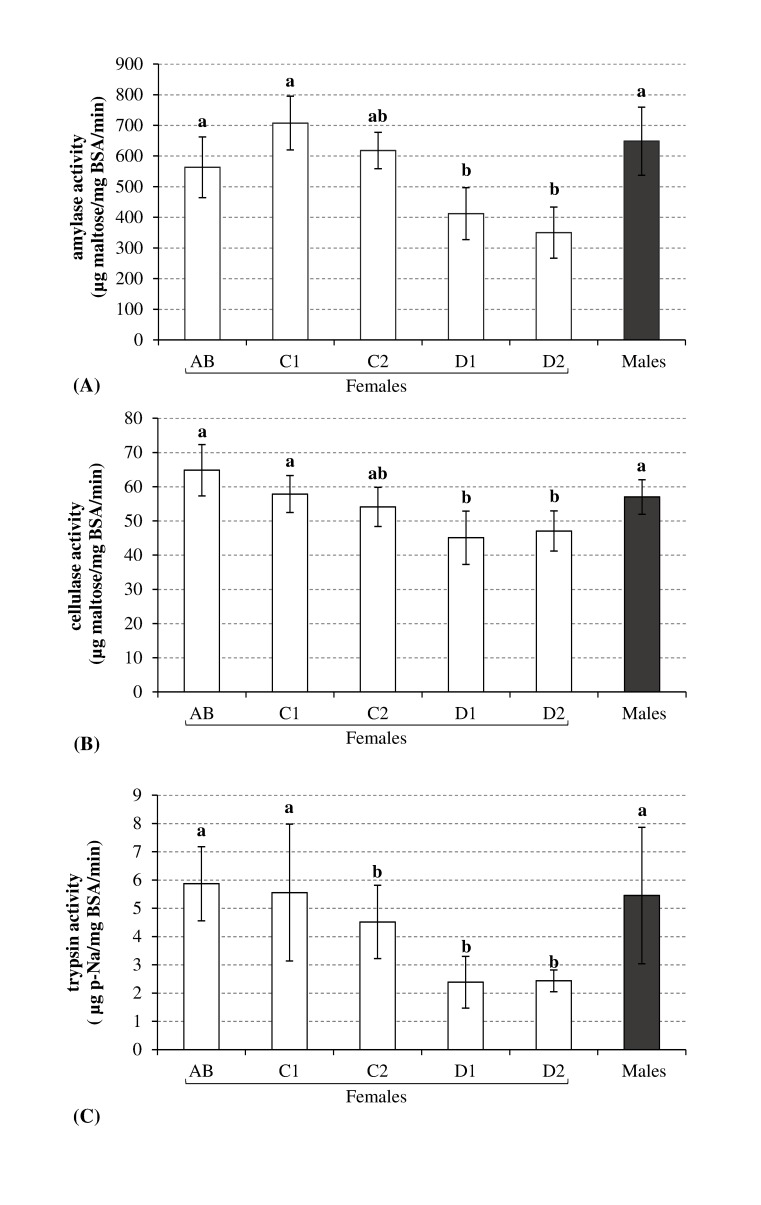
Digestive enzyme activities during each stage of the reproductive cycle in females and males (mean ± SD, n = 6). A: Amylase (mg maltose/mg BSA/min), B: Cellulase (mg maltose/mg BSA/min), C: Trypsin (µg p-Na/mg BSA/min). Histograms with the same letters are not statistically different (Kruskal-Wallis test: *p*<0.05).

The activities of amylase, cellulase, and trypsin (Figure 1) in female gammarids declined significantly (*p*<0.05) during the reproductive cycle. A similar pattern was observed in all cases, where the highest digestive enzyme activities were detected during stages AB and C1 compared with the other stages (D1 to D2). The second intermolt stage (C2) was an intermediate stage. The size of the reduction differed among the enzymes, i.e., the amylase, cellulase, and trypsin activities decreased to 51%, 31%, and 61% of the higher levels (AB or C1), respectively.

**Figure 2:**
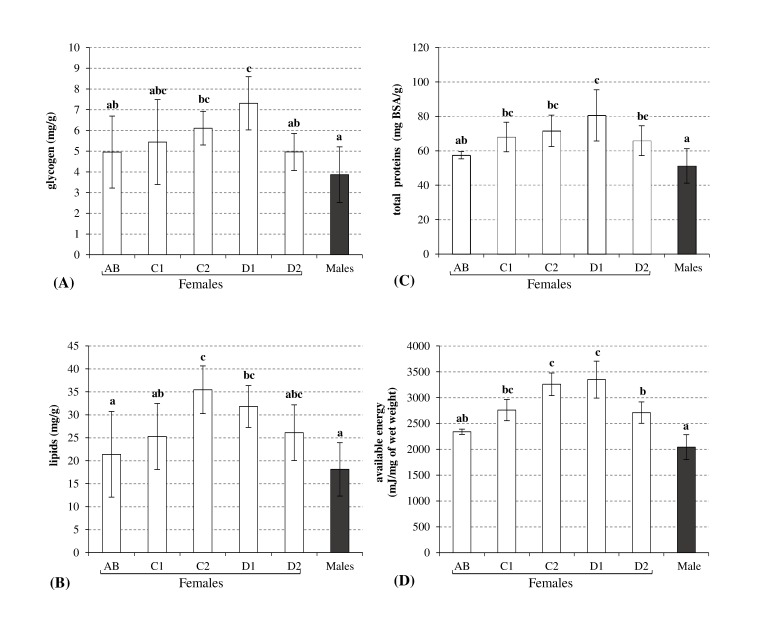
Energy reserve levels during each stage of the reproductive cycle in females and males (mean ± SD, n = 6). A: Glycogen (mg/g wet weight), B: Lipids (mg/g wet weight), C: Total proteins (mg BSA/g wet weight), D: Available energy (mg/mg wet weight). Histograms with the same letters are not statistically different (Kruskal-Wallis test: *p*<0.05).

The energy reserves of females were highly dependent on the molt stage and they followed a similar trend (Figure 2), except for the total free sugars (not shown), which did not differ among the reproductive stages. The reserves and Ea increased throughout the first four reproductive stages (postmolt AB, intermolt C1 and C2, and premolt D1), although the lipids increased from postmolt stage AB to intermolt C2. The average energy gains over the reproductive cycles of females were 32%, 40%, 29%, and 30% for glycogen, lipids, proteins, and Ea, respectively. In the second stage, a significant depletion of the Ea was detected in the final premolt stage (D2). The average energy losses at the end of the reproductive cycle were 32%, 26%, 18%, and 19% for glycogen, lipids, proteins, and Ea, respectively.

Molting and reproduction are considered to be fundamental events in the lives of crustaceans. Thus, the morphological and structural changes that occur during the molting cycle are reflected by physiological and biochemical variations [38]. In the present study, during the molt and reproductive cycle, the reserves (glycogen, lipids, and proteins) increased in female gammarids until the late intermolt stage (C2) (Figure 2), which correlated with the high digestive enzyme activities (Figure 1). During this first part of the reproductive cycle in female gammarids, the accumulation of energy may be necessary to respond to the growth of oocytes and to prepare for ecdysis, which are extremely costly events in terms of energy. In *Callinectes arcuatus,* Vega-Villasante et al. [39] also suggested that differences in the activities of the digestive enzymes were related to specific requirements for nutrients and energy during the stages of the molting cycle. Thus, the activities of digestive enzyme appear to be physiological responses that are adapted to energy needs.

Our findings are consistent with those of many other studies of crustaceans, which have highlighted the biochemical variations linked to molting. According to Martin [40], lipids, glycogen, and proteins are required to form the new cuticle. Therefore, lipids are stored continuously during the intermolt and early premolt periods in decapods and gammarids [40], [41]. In addition, the glycogen level increases in crustaceans between the late intermolt stage and the molt before decreasing thereafter, which shows that glycogen is a chitin formation precursor [42]. Our results also agree with other studies of the changes that occur during the crustacean reproductive cycle. Several studies suggest that breeding females accumulate and store lipids during the maturation of oocytes and vitellogenesis [31]. In some species, lipids account for 18–41% of the total ovarian dry mass at the end of ovarian maturation [21].

However, we observed that digestive enzyme activities declined from the end of the intermolt stage to ecdysis, despite the growing need for energy. The overall decrease in the activities of all digestive enzyme may be linked to the physiological (soft exoskeleton) and/or behavioral (amplexus) changes associated with poor access to food [43], [44]. In this context, it may be preferable for gammarids not to allocate energy to their digestive enzymes. Our results agree with other studies of the activities of digestive enzyme, particularly amylase and trypsin during the molting cycle in decapods, where decreases in the digestive enzyme activities occur during the late premolt stage [45]–[48]. In addition, Zilli et al. [49] detected changes in the cell type composition in the hepatopancreas of *Marsupenaeus japonicas*. During the molting cycle, F cells were implicated in enzyme synthesis and zymogen secretion declines in the premolt stages (D0 to D3). In addition to the molt cycle effect in female gammarids, the reproductive behavior during the premolt can disrupt the feeding activities of the female. Indeed, females must be accompanied by a male during amplexus, which begins toward the end of the female intermolt period and continues until ecdysis [50]. This precopulatory mate guarding behavior may impair the mobility and foraging capacity of the female. Thus, the females probably did not feed until the end of the cycle in the present study and all of their digestive activities declined. Amplexus and its effects on energy acquisition may explain the decline in biochemical compounds and Ea in the late premolt stage (D2). Indeed, a few days after the initiation of fasting, an organism has to draw on its energy reserves to ensure the completion of oogenesis, ventilation during egg incubation, and the formation of a new exoskeleton. Thus, the completion of the reproductive cycle depends greatly on food access during the cycle. In this context, Read and Caulton [51] also detected declines in lipids and proteins in *Panaeus indicus* during the last premolt stage, which may be attributable to the catabolic energy required for the forthcoming molt.

#### Males

Males were sampled during amplexus. The levels of digestive enzyme (Figure 1) were similar to those in females in stages AB and C1, i.e., the amylase, cellulase, and trypsin activities were 648 (±111.3) µg maltose/mg BSA/min, 56.9 (±5.06) µg maltose/mg BSA/min, and 5.4 (±2.41) µg p-Na/mg BSA/min, respectively. The levels of the reserves in males were also similar to those in females in the postmolt stage (AB). However, we found that the levels of all the parameters monitored in males (glycogen, lipids, proteins, and available energy) were always lower than those in females, except for the total free sugars (results not shown), which were the same as those in females.

It is interesting that the digestive enzyme activities in the males were equivalent to those in females during the intermolt stage. These digestive activities in males suggest indirectly that the males have the ability to feed during the precopulatory stage. This male feeding capacity during amplexus is also supported by Becker et al. [52]. Despite the presence of this feeding behavior followed by digestion (based on the enzyme activities), the individual males were physiologically exhausted. The energetic costs of precopulatory mate guarding may explain the low levels of glycogen, lipids, and proteins in males. Many previous studies suggest that high energy expenditure occurs in males during amplexus. Indeed, several behaviors are expected to be energetically costly, such as male-male competition [53], sexual conflict over the mate-guarding duration [54], and the cost of carrying a partner [55], [56].

### Effects of Food Starvation on Energy Metabolism Parameters

#### Digestive enzyme activities

**Figure 3:**
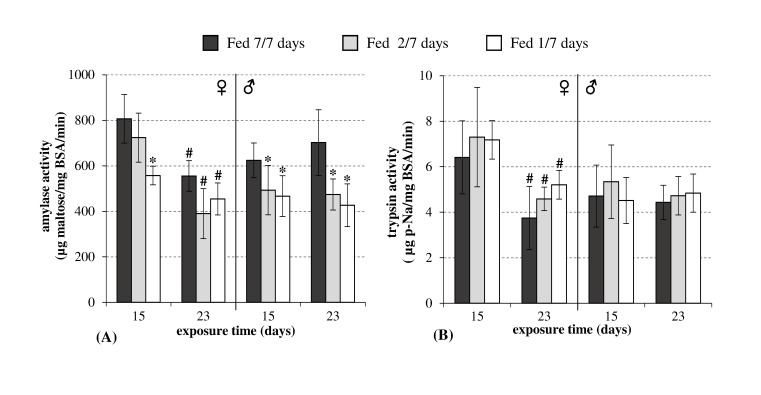
Digestive enzymes activities in *Gammarus fossarum* females (♀) and males (♂) exposed to three levels of diet stress (control, fed 7 days per week; fed 2 days per week; and fed 1 day per week) after 11 and 23 days (mean ± SD, n = 6). A: Amylase (mg maltose/mg BSA/min), B: Trypsin (µg p-Na/mg BSA/min). Stars indicate significant differences between the starved organisms and the fed control organisms (Mann-Whitney test: *p*<0.05). The hash (#) symbol indicates a significant difference between the two sampling time points for each diet condition tested (Mann-Whitney test: *p*<0.05).

The amylase activity decreased significantly (*p*<0.05) comparing to the control fed condition, except after 23 days in females (Figure 3A). By contrast, the trypsin activity levels (Figure 3B), were not affected by starvation in either gender.

The correlation between the decline in amylase activity and food starvation may reflect the implementation of an energy optimization strategy in a context of low dietary intake. Previous observations of the effects of starvation on carbohydrase enzymes in decapods agree with our results. Furthermore, a positive correlation between the amylase activity and ingested or assimilated food was demonstrated in different zooplankton species [57]. Kerambrun and Guérin [58] showed that the amylase activity of *Leptomysis lingvura* decreased after 8 days of starvation and remained at this level during subsequent weeks. Thus, the food quantity can have marked effects on the activities of digestive enzymes. The protease activity levels in crustacean decapods also appear to depend on food availability. For example, in the shrimp *Penaeus vannamei*, the hepatopancreas trypsin and chymotrypsin activities were 40–60% lower after 120 h of starvation [59]. Cuzon et al. [60] also showed that the trypsin activity declined in shrimps during starvation. By contrast, we detected no effects of food quantity on trypsin activity. This may be because *Tubifex* worms were not provided as a dietary supplement during this study of the effects of food starvation, which contrasted with their use as a dietary supplement during the acclimatization period. The gammarids were fed only with plant materials, with no animal protein.

Boucher et al. [61] studied the amylase and trypsin activities in *Temora stylifera* fed with a relatively low quantity of natural food, comparatively to organisms fed with abundant diet of monospecific phytoplankton. With a monospecific vegetable diet, the amylase activities were twofold higher whereas the trypsin activities were fivefold lower. These results demonstrate that the amylase and trypsin activities are modulated by food availability, and also by the protein and starch content of food. Similar observations were made by Van Wormhoudt et al. [62] in the shrimp, *Penaeus kerathurus*. Thus, the activities of digestive enzymes appear to be adjusted to the nature and the availability of food, thereby optimizing the efficiency of the digestive process. In addition, Garcia-Carreno and Hernandez [63] suggested the concept of enzymatic adaptation, where the activities of digestive enzyme vary according to food stresses, such as inadequate food or food containing anti-nutritional factors.

For both enzymes (Figures 3A and 3B), significant reductions (*p*<0.05) in their activities were observed only in females at the two sampling times under all feeding conditions (7/7, 2/7, and 1/7). These results are consistent with the conclusions of the previous part of this study regarding the effects of the reproductive cycle on energy metabolism responses.

At the start of this study, the females were at stage AB, but after 11 and 23 days they were at stages C1 and D1, respectively. The decrease between day 11 and day 23 was consistent with the decline observed during the female reproductive cycle between stages C1 and D1 (see below). In addition, the absence of a gradient in the digestive enzyme activities after 23 days of food stress could be explained by behaviors (precopulatory mate-guarding) related to the reproduction process. In stage D1, the females did not feed in any of the experimental conditions (fed, 7/7, 2/7, or 1/7; see below). The stable digestive enzyme activities of males between day 11 and day 23 suggest a lower effect of the reproductive cycle in males compared with that in females.

#### Energy reserves

**Figure 4:**
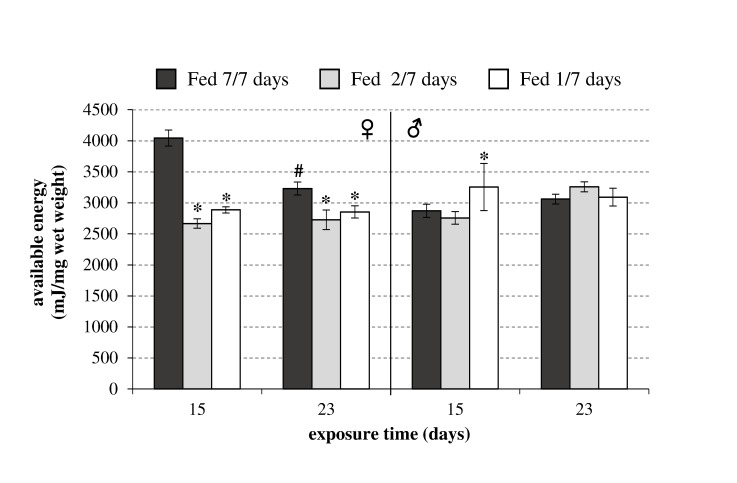
Available energy (mJ/mg wet weight) in *Gammarus fossarum* females and males exposed to three levels of diet stress (control, fed 7 days per week; fed 2 days per week; and fed 1 day per week) after 11 and 23 days (mean ± SD, n = 6). Stars indicate significant differences between the starved organisms and the fed control organisms (Mann-Whitney test: *p*<0.05). The hash (#) symbol indicates a significant difference between the two sampling time points for each diet condition tested (Mann-Whitney test: *p*<0.05).

A significant effect of food deprivation (*p*<0.05) was detected in the female energy reserves after 11 and 23 days (Figure 4). After 11 days, the females fed 1/7 and 2/7 had Ea reductions of 28.6% and 34%, respectively, compared with the control. These Ea reductions were less than those of the control after 23 days for females fed 1/7 and 2/7, with decreases of 11.6% and 15.5%, respectively. In diet stress conditions, the Ea in females was lower than that in the control state. By contrast, no effect of starvation on Ea was observed in the males.

Based on these results in males, we hypothesize that: (i) low quantities of food (fed 1/7) were sufficient to meet the basal energy needs of males, or (ii) all of the diets tested were not adapted to the nutritional requirements and all of the organisms were depleted. However, the Ea in males supports the first hypothesis. After feeding with leaves and worms in the acclimatization conditions (Figure 2), the males had average Ea values of 2000 mJ/mg (wet weight). In the diet stress conditions, the males had Ea values between 2700 and 3200 mJ/mg (wet weight), which did not suggest an energy metabolism impairment.

In females, however, we observed significant Ea declines in starved organisms (fed 1/7 or 2/7) compared with those fed 7/7. These results highlight the different metabolic needs of males and females. Females appear to need more energy to complete their reproductive cycles. Thus, diet stress might have affected the Ea level at each sampling time point and throughout the reproductive cycle. Disturbances of the energy balance in females could affect their reproductive success. Therefore, it would be useful to compare the oocyte number and their embryonic development in each diet condition to assess the effects of food starvation at the individual level.

## Conclusion

The present study assessed the effects of physiological parameters (gender and female reproductive cycle) and food availability on two energy metabolic responses (digestive enzyme activities and Ea) in *G. fossarum*. This ecophysiological study of *Gammarus fossarum* provides novel biological information, which will facilitate a better understanding of the physiological and biochemical responses of gammarids. These data could also help to understand energy metabolism in gammarids. We determined the effects of the reproductive cycle on the activities of digestive enzyme and Ea in females, and highlighted the more stable responses found in males. In female gammarids, two hypotheses may explain the fluctuations in their digestive activities and energy storage levels: (i) physiological events (molt, oogenesis, and spawning) and (ii) reproductive behavior (amplexus). We also demonstrated the effects of food on the digestive activities and energy outcomes in both genders. First, the effect of the food quantity was illustrated by higher amylase activity levels when more food was available. Second, the effect of food quality was highlighted by the stable trypsin activity level in organisms fed a monospecific vegetable diet with no protein supplements. Given the responses of these biomarkers, we suggest that the experimental designs used in field studies should aim to reduce the effects of these parameters, which are possible confounding factors in active biomonitoring. To limit the effects of physiological processes (such as molt and reproduction) on the biological responses being studied, we advocate the use of calibrated males. In the field, the possible differences in the trophic levels among study sites could be limited by using caging approaches, where the organisms are fed *ad libitum*. In conclusion, our results confirm that males fed *ad libitum* can be used in different active biomonitoring studies [9], [10], where their metabolic responses can serve as biomarkers.
